# Population Genomic Analysis Reveals Highly Conserved Mitochondrial Genomes in the Yeast Species *Lachancea thermotolerans*

**DOI:** 10.1093/gbe/evu203

**Published:** 2014-09-11

**Authors:** Kelle C. Freel, Anne Friedrich, Jing Hou, Joseph Schacherer

**Affiliations:** Department of Genetics, Genomics and Microbiology, University of Strasbourg/CNRS, UMR7156, France

**Keywords:** mitochondrial genome, intraspecific diversity, selection, genome evolution

## Abstract

The increasing availability of mitochondrial (mt) sequence data from various yeasts provides a tool to study genomic evolution within and between different species. While the genomes from a range of lineages are available, there is a lack of information concerning intraspecific mtDNA diversity. Here, we analyzed the mt genomes of 50 strains from *Lachancea thermotolerans*, a protoploid yeast species that has been isolated from several locations (Europe, Asia, Australia, South Africa, and North / South America) and ecological sources (fruit, tree exudate, plant material, and grape and agave fermentations). Protein-coding genes from the mtDNA were used to construct a phylogeny, which reflected a similar, yet less resolved topology than the phylogenetic tree of 50 nuclear genes. In comparison to its sister species *Lachancea kluyveri*, *L. thermotolerans* has a smaller mt genome. This is due to shorter intergenic regions and fewer introns, of which the latter are only found in *COX1*. We revealed that *L. kluyveri* and *L. thermotolerans* share similar levels of intraspecific divergence concerning the nuclear genomes. However, *L. thermotolerans* has a more highly conserved mt genome with the coding regions characterized by low rates of nonsynonymous substitution. Thus, in the mt genomes of *L. thermotolerans,* stronger purifying selection and lower mutation rates potentially shape genome diversity in contract to what was found for *L. kluyveri*, demonstrating that the factors driving mt genome evolution are different even between closely related species.

## Introduction

Within an organism, differential rates of evolution exist in both the nuclear and the organelle genomes. Mitochondrial (mt) DNA, in particular, has provided an interesting framework to examine genome dynamics. Among a variety of model systems ranging from *Caenorhabditis elegans* to *Drosophila melanogaster*, mutation rates in the mt genome are consistently higher than in the nuclear genome (Denver 2000; Haag-Liautard et al. 2008). This is also the case in yeast (Lynch et al. 2006, 2008), and has been explained by the presence of more reactive oxygen species, increased replication rates and a lack of sufficient repair proteins (Solieri 2010) in the mt DNA. However, it is evident that purifying selection plays an important part in mt genome maintenance (Denver 2000; Jung et al. 2012). Thus, although elevated mutational pressures characterize the evolution of mt genomes, purging selection guarantees coding sequence functionality. The extent to which selection shapes genome evolution of different yeast species has not been adequately explored.

The availability of complete mt genomes in more than 40 lineages of the *Saccharomycotina* (Solieri 2010; Gaillardin et al. 2012) has provided insight concerning the evolution of an entire phylum. Among these genomes, there is a core set of conserved genes, including three subunits of ATP synthetase (*ATP6, ATP8*, and *ATP9*), apocytochrome b (*COB*), three subunits of cytochrome oxidase (*COX1, COX2*, and *COX3*), the large and small rRNA genes, and a complete set of tRNA genes. Other genes, in particular those encoding the subunits of NADH dehydrogenase, *VAR1* and the RNA subunit of the mitochondrial RNAse P (*RPM1*) (Jung et al. 2009) are not present in all mt genomes. Additionally, mtDNA organization, architecture, and size are different among species, with mtDNA ranging from 11 to 85 kb in length in *Hanseniaspora uvarum* and *Saccharomyces cerevisiae,* respectively (Foury et al. 1998; Pramateftaki et al. 2006). Yeast mtDNA can be rich in introns and long noncoding regions, which account for these differences in size (Friedrich et al. 2012; Gaillardin et al. 2012). The gene order among mtDNA in the *Saccharomycotina* is highly variable and the result of multiple rearrangements (Solieri 2010). It is clear that the genetic architecture can also be quite diverse, as some genomes are organized as linear concatemers whereas others are circular monomers (Solieri 2010). Research concerning closely related lineages demonstrates, however, that on a shorter evolutionary timescale, gene content, and synteny are generally conserved across species whereas intron content and intergenic regions are not (Kosa et al. 2006; Friedrich et al. 2012; Gaillardin et al. 2012).

The first study to focus on mt genome diversity in multiple strains of a single yeast species was completed with *Lachancea kluyveri* (Jung et al. 2012). This work found that the mt genomes ranged in size from 50.1 to 53.7 kb, due to changes in intron content, while, all protein-coding genes were syntenic. Most interestingly, the genomes were extremely polymorphic, especially in the intergenic regions, suggesting that these regions have evolved rapidly in *L. kluyveri*. Although careful examination of *L. kluyveri* was a step toward understanding mtDNA evolution, the extent to which these results mirror intraspecific mt genome evolution in other clades was unknown.

Recently, multiple species from the *Lachancea* genus were analyzed to explore interspecific variation across closely related lineages (Friedrich et al. 2012). The mt genomes share a similar architecture as well as a syntenic organization of genes. Intriguingly, although the protein sequences are remarkably alike, the intergenic regions are extremely variable, indicating that most mt genes are under purifying selection across the entire *Lachancea* genus. Both the nuclear and mt genomes of the *Lachancea thermotolerans* type strain CBS 6340^T^ were previously sequenced (Talla et al. 2005). This isolate was originally obtained from fermenting plum jam in 1932 and is commonly found in association with fruit, *Drosophila* sp. and other insects. Analysis of the *Lachancea* genus revealed that *L. kluyveri* and *L. thermotolerans,* respectively, represent both the largest (51.5 kb) and smallest (23.6 kb) genomes in the clade. Apart from the discrepancy in size, the GC content, the percentage of the genome dedicated to intergenic regions, and the number of introns also distinguish these species. Thus, analysis of the intraspecific diversity within *L. thermotolerans* is key to determine if the patterns of divergence in the intergenic and coding regions in *L. kluyveri* reflect those in other yeast species.

Here*,* we sequenced the genomes of 50 *L. thermotolerans* isolates representing a range of locations (Europe, Asia, Australia, South Africa, South America, and North America) and substrates (fruit, tree exudate, plant material, and grape and agave fermentations), in order to investigate intraspecific species diversity. We used the previously available and completely assembled mt genome of *L. thermotolerans* CBS 6340^T^ as a reference. The genome of the type strain is 23,584 bp and shares all genes with *L. kluyveri* (Jung et al. 2012). From a phylogenetic tree constructed with the concatenated CDS of the 50 mt genomes, eight strains were chosen for de novo mt genome assembly. In comparison to *L. kluyveri*, the intergenic regions are smaller and only the *COX1* gene harbors introns. Interestingly, mt genomes among *L. thermotolerans* isolates are highly conserved, have low d*N*/d*S* values across all protein-coding genes and appear to be under more extreme levels of purifying selection than in *L. kluyveri.* In addition, an analysis of 50 randomly chosen nuclear genes revealed that *L. thermotolerans* and *L. kluyveri* share similar levels of divergence at the nuclear genome level. This study is an important step in elucidating intraspecific mtDNA diversity in yeast, and demonstrates that differential selection pressures can act on mt genomes even among closely related species.

## Materials and Methods

### *Lachancea thermotolerans* Isolates and DNA Preparation

The 50 strains sequenced in this study were obtained from culture collections and multiple generous laboratories. The isolates chosen for sequencing represent diverse geographical locations (Europe, Asia, Australia, South Africa, South America, and North America) and ecological niches (fruit, tree exudate, plant material, and grape and agave fermentations) (supplementary table S1, Supplementary Material online). Yeast cell cultures were grown overnight at 30°C in 20 ml of YPD medium to early stationary phase before cells were harvested by centrifugation. Total genomic DNA was subsequently extracted using the MasterPure Yeast DNA purification kit (Cat No MPY80200) according to the manufacturer’s instructions.

### Sequencing of *L. thermotolerans* Genomes and Detection of Polymorphisms

Genomic Illumina sequencing libraries were prepared with a mean insert size of 500 bp. The 50 libraries were multiplexed in two Illumina HiSeq 2000 lanes and subjected to paired-end sequencing (2 × 100 bp). The reads were then mapped with the Burrows–Wheeler Aligner (version 0.7.0) (Li and Durbin 2009) to the CBS 6340^T^ nuclear and mt reference genomes (Talla et al. 2005; Souciet, J-L et al. 2009), allowing eight mismatches and two gaps. Single nucleotide polymorphisms (SNPs) and small indels were called using Samtools (version 0.1.18) (Li and Durbin 2009).

### Nucleotide Diversity

An estimation of π, the average pairwise nucleotide diversity, was calculated with Variscan (Hutter et al. 2006). This allowed for the characterization of nucleotide diversity within coding sequences of the mitochondrial and nuclear genomes for both *L. thermotolerans* and *L. kluyveri*. In order to determine if the difference in diversity found within the two species was statistically significant, and *P* values were calculated using a Student *t*-test on a set of 100 subsamples from the data set. Each sample was composed of 75% of the total number of strains available, all of which were randomly selected.

### Phylogenetic Analyses of Mitochondrial and Select Nuclear Genes

For each strain, the sequences of the eight protein-coding genes (*ATP6*, *ATP8*, *ATP9, COB*, *COX1*, *COX2*, *COX3,* and *VAR1*) were reconstructed based on the SNP data generated from the polymorphism detection step described above. These nucleotide sequences were automatically aligned with MAFFT (Edgar 2004) and manually inspected before concatenation. Based on the resulting 6,612 positions, phylogenetic relationships among *L. thermotolerans* strains were analyzed with PhyML using the maximum-likelihood method (with the Hasegawa-Kishino-Yano 85 substitution model) (Guindon and Gascuel 2003). Bootstrap analyses (100 replicates) were completed to assess the confidence level of each node in the phylogeny. The same strategy was also implemented for the construction of a *COX1* phylogeny as well as a phylogenetic tree based on 50 concatenated nuclear genes (supplementary table S2, Supplementary Material online) that had orthologs in *L. kluyveri*. In total, the 50 genes represented 66,358 sites. The identification of these orthologous genes was based on data previously generated by Fischer G and Neuvéglise C (unpublished data). None of the genes included were functionally annotated.

### Mitochondrial Genome Assembly and Annotation

The reads that did not map to the nuclear reference genome of CBS 6340^T^ were used for de novo construction of the mt genomes. For each strain, automated assemblies were completed with SOAPdenovo2, version 2.04 (Luo et al. 2012) using the two Kmer size (soapdenovo63mer with option –K 63 and soapdenovo127mer with –K 75). For eight strains (CBS1877, DBVPG 2551, DBVPG 3466, DBVPG 3469, DMKU-RK361, IY 05-6-6-2-3-3, NRRL Y-27937, UWOPS 83-1097.1), we identified mt scaffolds by performing similarity searches with the BLAST suite of programs (Altschul et al. 1997). Between 3 and 126 scaffolds were identified in each assembly. Based on sequence overlap, these assemblies were manually refined to obtain a single scaffold of each mt genome.

The CBS 6340^T^ EMBL annotations were automatically transferred to each mt genome sequence generated with RATT (Otto et al. 2011), and were then manually edited by direct sequence inspection. Sequences were submitted to the EMBL and accession numbers (LK392296–LK392303) were obtained for strains CBS 1877, DBVPG 2551, DBVPG 3466, DBVPG 3469, DMKU-RK361, IY 05-6-6-2-3-3, NRRL Y-27937, and UWOPS 83-1097.1, respectively.

### Analysis of Selection Pressure and Calculation of d*N*/dS Ratios

The d*N*/d*S* ratios were determined using the CODEML model in the PAML package version 4.4 b (Yang 2007) using a tree-based maximum-likelihood method. In summary, coding sequence multialignments were generated for each of the eight protein-coding genes from all *L. thermotolerans* mt genomes, as described previously. Based on these alignments, neighbor-joining trees were constructed with ClustalX (Larkin et al. 2007) and then labeled manually. Estimates of the d*N*/d*S* ratios were then calculated for each gene.

## Results and Discussion

In an attempt to further elucidate intraspecific mt genome diversity in yeast, we obtained complete mtDNA sequences from 50 isolates of the yeast species *L. thermotolerans.* These strains represented diverse ecological niches and various locations (supplementary table S1, Supplementary Material online). This is only the second study of its kind to focus on intraspecific mt diversity, and, to our knowledge, presents the most extensive data set available for one species. Ultimately, we procured a valuable group of sequences for the expansion of intraspecies mt genome comparisons among clades of the *Lachancea* genus.

### A Model System for the Analysis of Inter and Intraspecific Diversity: Sister Species of the *Lachancea* Genus

Previous to this work, only one study assessed the intraspecies mt diversity within a single yeast lineage, specifically, among 18 isolates of *L. kluyveri* (Jung et al. 2012). This species is most closely related to *L. thermotolerans* and the mt genomes share a variety of features (Talla et al. 2005). Of the 35 genes found in both species, eight encode proteins whereas the remaining 27 are noncoding RNA genes. All genes are syntenic and transcribed from a single DNA strand (Jung et al. 2009). The *L. thermotolerans* CBS 6340^T^ mt genome is 23,584 bp in length with a GC content of 24.8% (supplementary fig. S1, Supplementary Material online), whereas the average *L. kluyveri* mt genome is approximately twice this size at 51 kb and has a much lower GC content of 15%. This dramatic difference is due to changes in the number and size of the introns (Jung et al. 2009). The intergenic regions of the type strain CBS 6340^T^ mt genome comprise 24.2% of the total sequence, all three introns are found in *COX1*, and have a total combined length of 4,320 bp. This previous knowledge provided a framework for our study, and allowed us to focus on examining *L. thermotolerans* intraspecific mt diversity.

### Sequencing of an Unprecedented Data set, 50 Mitochondrial Genomes of *L. thermotolerans*

The 50 complete mt genomes of *L. thermotolerans* strains used in this study were obtained on an Illumina HiSeq 2000 platform. A total of 440 Gb of high-quality sequence data was generated with a resulting mean coverage of 65 × for the nuclear and 2,130 × for the mt genomes. The mean number of SNPs in the coding regions, intergenic regions and introns were 6 (0–15 out of 6,612 bp), 63 (1–158 out of 5,701 bp), and 48 (0–114 out of 4,326 bp), respectively (supplementary table S3, Supplementary Material online). All genomes shared a core set of eight genes also found in *L. kluyveri*: *ATP6, ATP8, ATP9, COX1, COX2, COX3, COB,* and *VAR1*. This sequence data represents an ideal tool for the investigation of intraspecies mtDNA diversity in a single yeast clade.

### Phylogeny and Genetic Diversity of *L. thermotolerans* Mitochondrial and Nuclear Genomes

**Fig. 1.—:**
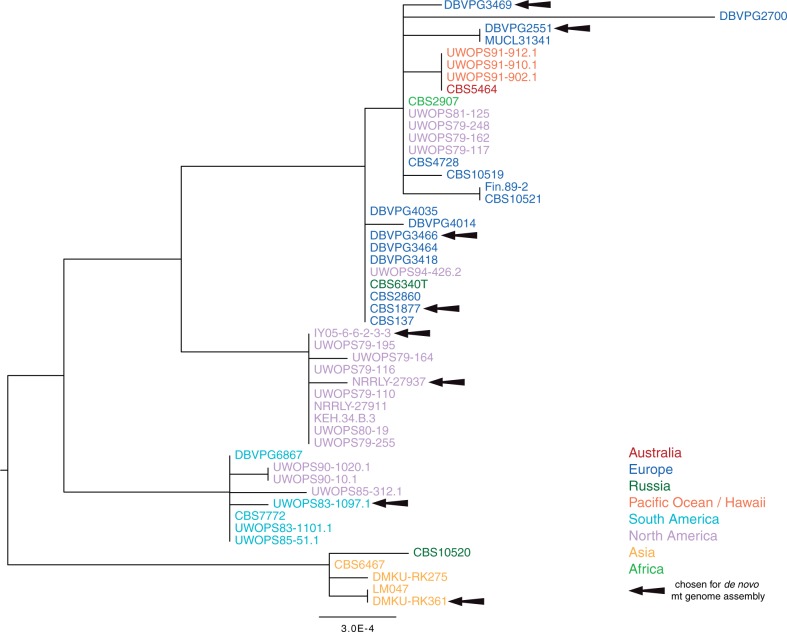
Phylogenetic tree constructed with PhyML of the concatenated coding sequences for *ATP6-ATP8-ATP9-COX1-COX2-COX3-COB-VAR1*, extracted from the 50 genomes. Colors correspond to the geographic location from which strains were isolated. Black arrows highlight the strains for which de novo assembly of the mitochondrial genomes was completed. The CDS were aligned independently for each gene, a total of 6,612 bp per strain were used to construct the tree as well as 100 bootstrap replicates.

To illustrate the intraspecific mt genome evolution in *L. thermotolerans*, a phylogenetic tree was constructed using the concatenated protein-coding sequences from the mt genome (*ATP6-ATP8-ATP9-COX1-COX2-COX3-COB-VAR1*). The phylogeny was built with PhyML using data from all 50 genomes and 100 bootstrap replicates were run. This phylogenetic tree (fig. 1) is composed of 5 clades, one of which consists exclusively of strains from North and South America whereas another is only isolates from Asia. The remaining three clades are composed of isolates with mixed geographic origins. For example, one cluster includes strains from North America, South Africa, and Europe. These results are not as definitive as those for *L. kluyveri*, where the phylogeny clearly supported the hypothesis that the evolution of the species was linked to geographical isolation in North America, Europe, and Asia (Jung et al. 2012). Additionally, isolates from substrates including tree exudate and fruit are spread across the clades of the mt phylogeny. Thus, as was previously found with *L. kluyveri*, the ecological origins of the isolates do not appear to have any influence on the resulting clustering of *L. thermotolerans*. Previous work on *S. cerevisiae* demonstrated that strains from the same ecological origin cluster together phylogenetically (Liti et al. 2009; Schacherer et al. 2009), whereas studies on *Saccharomyces paradoxus* have instead found evidence that clades tend to correspond to the geographical location isolates were obtained from.

**Fig. 2.—:**
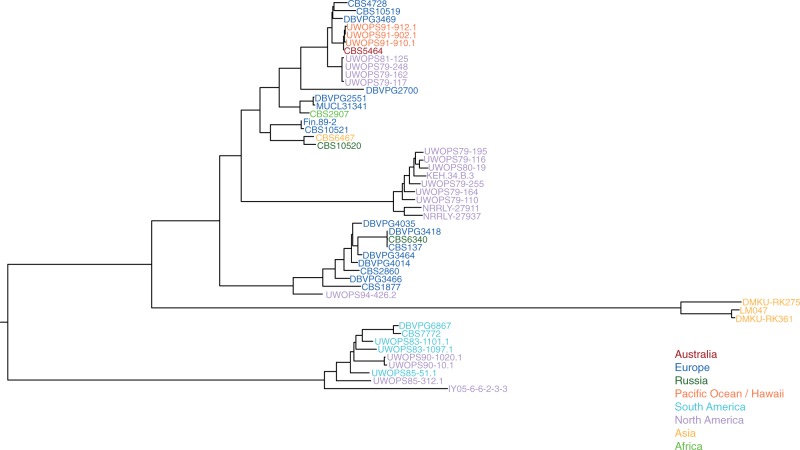
Phylogenetic tree constructed with PhyML of 50 random genes chosen from the nuclear genome of the *L. thermotolerans* reference strain (CBS 6340^T^). All 50 genes were obtained and concatenated for each of the 50 isolates represented in the phylogeny. A total of 66,358 polymorphic sites were used in tree construction. See supplementary table S2, Supplementary Material online, for a list of all genes included as well as their respective positions in the concatenated alignment.

Analysis of mt genomes is a useful method to investigate evolution among closely related isolates. It is essential to also utilize nuclear sequence data in order to clarify relationships within strains and between species. In order to compare the phylogeny of strains obtained with the concatenated mt protein-coding sequences to that of the nuclear genome, 50 genes (supplementary table S2, Supplementary Material online) were randomly selected for the construction of an additional phylogenetic tree (fig. 2). The *L. thermotolerans* nuclear phylogeny is composed of six clades, revealing a higher level of resolution than is illustrated with the mt genome. Of the clusters, three are loosely correlated to geographic location (Asia, North America, and North/South America clades), while the two most closely related clades are composed of samples from various distant locations (including Italy, the Uunited States and Australia). The nuclear phylogeny (fig. 2) roughly reflects the same clades found in figure 1 with the exception of three isolates, which demonstrated discordant phylogenies. Some incongruence was expected as previous studies found that data sets composed of linked genes, such as those in the mt genome, are unreliable for establishing phylogenetic hypotheses (Rokas et al. 2003). The inconstant clustering of these three isolates could be linked to recombination events with other strains in the history of the lineages. Thus, to obtain an accurate tree, it is important to use data sets composed of a minimum of 20 independently evolving genes that are distributed throughout the genome (Rokas et al. 2003). Overall, however, the general pattern of clustering was similar in the two phylogenies.

In addition to comparing the phylogenetic clustering between the mt and nuclear genomes of *L. thermotolerans*, we also analyzed the genetic diversity in these two data sets by inferring the polymorphic sites across the coding regions of the sequences. The mt genomes had incredibly low levels of intraspecific divergence (π = 0.0014) in comparison to the nuclear genome (π = 0.012). These rates were unexpectedly low as the previous study on *L. kluyveri,* revealed the presence of highly polymorphic mt genomes with particularly variable intergenic regions (π = 0.009). The mt genetic diversity between these two closely related species is significantly different according to the Student *t*-test results (*P* < 0.001). These different patterns of diversity led us to determine the range of sequence divergence between the nuclear genomes of these two sister lineages.

In order to compare the evolutionary history and nucleotide divergence between the two *Lachancea* species, a concatenated dataset corresponding to the same 50 nuclear genes chosen for *L. thermotolerans* was also constructed using the orthologous genes in *L. kluyveri*. The nuclear genomes of the *L. thermotolerans* isolates have similar levels of divergence (π = 0.012) in comparison to *L. kluyveri* (π = 0.014). This analysis clearly demonstrated that the two species are similar in regard to the evolution of their nuclear genomes. This was an important step in investigating intraspecific genome diversity in *L. thermotolerans*; however, to obtain a better picture of genome evolution within the species, eight representative genomes were chosen for de novo assembly and further study.

### De Novo Assembly of *L. thermotolerans* Mitochondrial Genomes

Although data generated for the 50 genomes was informative in order to obtain a better view of genome evolution, a subset of mt genomes were chosen for full assembly. Previous work found high levels of intraspecific mt genetic diversity within *L. kluyveri* to be in the intergenic regions (Jung et al. 2012). Indeed, in the coding sequence, the SNP density ranged from 0 to 13.96 SNPs/kb, while in the intergenic regions it was 0.31–82.7 SNPs/kb. Therefore, to have a global view of the diversity within *L. thermotolerans*, we completely assembled eight genomes that included at least one representative from each of the clades in figure 1. The reads selected each generated a single scaffold for the mt genomes and the reference genome CBS 6340^T^ was used for all sequence annotation.

The complete mt genomes ranged in size from 21,893 to 24,992 bp, and the GC % from 23.9 to 24.9% (table 1). Analysis revealed that all eight genomes are extremely well conserved. Additionally, across the genomes, there is a lack of rearrangements, which was also the case previously found among the isolates of *L. kluyveri* (Jung et al. 2012) and across multiple species within the *Lachancea* genus (Friedrich et al. 2012). Intraspecific variation of mt genomes in *L. thermotolerans* is low in the intergenic sequences and even lower in the coding regions. Out of 6,612 sites, on average, 6.4 (0 -15) SNPs were detected in the CDS, and 60.8 (1–158) SNPs in the intergenic regions.

**Table 1:** Summary of the Data Available for the Assembled Genomes

Strain	Scaffold Size	Scaffold Size Without N	Intergenic Size	Number of Introns	Intron Size	GC%	SNPs				
							Total	In CDS	In Intergenic	In Intron	In rRNA
CBS 6340^T^	23,584	23,584	5,701	3	4,326						
CBS 1877	23,611	23,574	5,733	3	4,333	24.9	20	0	14	4	2
UWOPS_83-1097.1	23,167	23,161	5,407	3	4,209	24.4	259	13	118	97	31
DMKU-RK361	25,121	25,121	5,761	4	5,822	23.9	286	15	158	75	38
NRRL_Y-27937	21,893	21,879	5,531	2	2,820	24.4	192	9	74	85	24
IY_05-6-6-2-3-3	24,693	24,692	5,501	4	5,648	24	206	8	70	102	26
DBVPG 2551	23,576	23,576	5,714	3	4,314	24.8	49	3	28	16	2
DBVPG 3466	23,592	23,575	5,716	3	4,327	24.9	3	0	2	1	0
DBVPG 3469	24,992	24,986	5,621	4	5,822	24.7	46	3	22	15	6

The assembly of the eight mt genomes provided a global view of the intergenic variability and intron content among *L. thermotolerans* isolates. It was previously established that intergenic content of mt genomes varies between closely related yeast (Jung et al. 2010; Gaillardin et al. 2012) and even within a single species (Jung et al. 2012). This is indeed the case between *L. thermotolerans* and *L. kluyveri* where intergenic sequences comprise on average 24% and 58% of the genome, respectively. The smaller intergenic regions in *L. thermotolerans* provide fewer targets for mutation, a potential explanation for a lower mutation rate. Interestingly, differences among strains of *L. thermotolerans* are almost exclusively linked to *COX1*, the only gene with introns.

### Low d*N*/dS Values in *L. thermotolerans* mt Genomes Possibly Driven by Purifying Selection and/or Low Mutation Rates

To elucidate mt intraspecific variability in *L. thermotolerans*, sequence diversity was compared among the 50 isolates. The total size of the CDS for all *L. thermotolerans* is 6,612 bp and there are 51 segregating sites (0.7% of the CDS). To further explore how purifying selection might affect the mt genomes, the ratio of nonsynonymous (d*N*) to synonymous (d*S*) substitutions was calculated. As mentioned previously, in *L. thermotolerans*, between 0 and 15 SNPs were detected in the coding genes, which did not include any nonsynonymous changes with the exception of the *VAR1* gene (9). Additionally, in two strains an indel of 3 bp was also identified in *VAR1*. Essentially only synonymous substitutions were present.

**Fig. 3.—:**
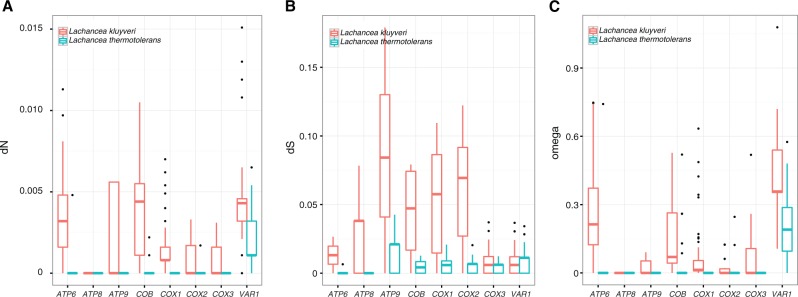
Box-plot comparisons of the dS (*A*) and dN values (*B*) and (*C*) as well as the dN/dS ratio [ω] which were estimated in the eight protein-coding mt genes and based on pairwise alignments. The values determined for *L. kluyveri* are shown in red, whereas those for *L. thermotolerans* are in blue. All genes appear to be under purifying selection or are subject to low mutation rates, and in all cases except *VAR1*, the diversity within *L. kluyveri* is significantly elevated in comparison to *L. thermotolerans*. Interestingly, *VAR1* displays the highest ω value for both species.

This average d*N*/d*S* value (ω) was determined for each of the coding regions. As predicted, all values are incredibly low across these CDS (fig. 3) and reveal that the mt genome of *L. thermotolerans* is possibly subject to extreme levels of purifying selection and/or incredibly low mutation rates, even in comparison to *L. kluyveri*. All ω values for the eight genes were below one, and only the *VAR1* gene had a d*N*/d*S* value over zero (median values of 0.2). The *VAR1* gene also had the highest values (median value of 0.35, supplementary fig. S2, Supplementary Material online) in the *L. kluyveri* study compared with the other genes which all had average values of 0.21 and lower, suggesting that *VAR1* is under different functional constraints than the other protein-coding genes in the mtDNA. An analysis of 1,125 bp of the *VAR1* sequence revealed that this gene roughly reflects the same phylogeny as that of the complete set of contacted genes in figure 1 (supplementary fig. S2, Supplementary Material online). In the coding regions of *L. kluyveri,* a total of 208 segregating sites out of a total 6,591 bp (3.2% of the CDS) were identified among the 18 isolates analyzed, which is much more than was found in *L. thermotolerans* (51 out of 6,612 bp). Of these positions, 171 were nonsynonymous SNPs and 930 were synonymous SNPs, demonstrating that purifying selection likely plays a key role in removing nonsynonymous changes. Although both data sets indicate that strong purifying selection and low rates of mutation influence mt genome evolution it is clearly a stronger force in *L. thermotolerans*. In addition to analyzing the conserved coding regions, we also investigated the intron content diversity among the eight de novo assembled genomes of *L. thermotolerans*.

### Intron Content Diversity among the Fully Assembled Genomes

**Fig. 4.—:**
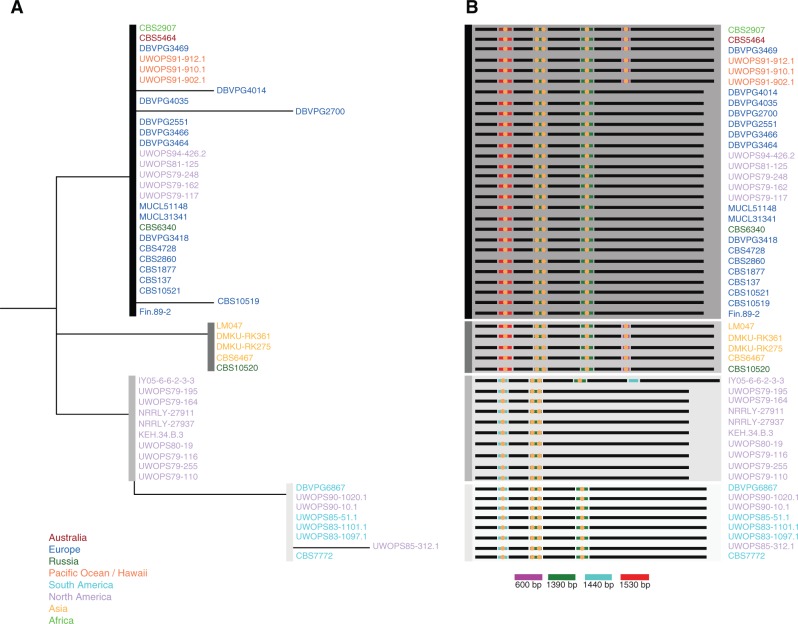
(*A*) Phylogenetic tree constructed of the *COX1* gene from the *L. thermotolerans* mt genomes constructed with PhyML. (*B*) A schematic illustration of the main differences among the assembled genomes in regard to intron organization in *COX1*. In the diagram of the *COX1* gene, circles indicate regions that encode endonucleases belonging to the LAGLIDADG family of group I introns. The length of each intron is indicated in base pairs and color coordinated to the key shown in the figure.

It has been established that mt intron content is variable between closely related species (Gaillardin et al. 2012). Our data confirm this finding, as across 50 isolates of *L. thermotolerans*, the *COX1* subunit is the only gene with introns. This gene contains between 2 and 4 introns, and is highly conserved (fig. 4), with only 0–5 SNPs in the coding regions among the 50 genomes. The introns in the *COX1* gene encode endonucleases which belong to the LAGLIDAGD family of group I introns (Haugen et al. 2005). The main differences among the *L. thermotolerans* mt genomes are indeed linked to the intron content of this gene (fig. 4). Although it is well conserved among all isolates, there are clusters with multiple intron insertion patterns. Interestingly, a recent study found that one of the insertions in *L. thermotolerans* closely resembles a translational bypassing element, which potentially contributes to diversification of proteins (Lang et al. 2014). However, it does not seem to have had this affect in light of the high sequence homology of isolates in this study.

Among genomes in the *Lachancea* genus, the intron content of the *COB*, *COX1,* and *LSU* genes is variable (Friedrich et al. 2012). In fact, the most pronounced differences are in the number of introns, which range from 2 in *Lachancea nothofagi* to 6 in *Lachancea meyersii*. Comparative analysis across seven *Lachancea* species, concluded that ultimately, mt genome evolution is mostly related to intron motility and intergenic region variation. This finding was reported in other instances as well, both between closely and distantly related species (Bouchier et al. 2009; Gaillardin et al. 2012).

## Conclusion

This is only the second study to thoroughly examine the intraspecific mt diversity within a single yeast species. In comparison to what was previously found, isolates sampled from *L. thermotolerans* harbor a set of mt genomes that are both syntenic and extremely conserved. The fact that the diversity among mtDNA in *L. thermotolerans* is different in comparison to the closely related *L. kluyveri*, but similar at the nuclear DNA level indicates that selection pressures are acting differentially on these two lineages. If they indeed occupy distinct niches, it is possible that they have varying respiration rates, which could lead to unequal dependence on the mitochondria. The ecological niche that *L. thermotolerans* inhabits might, in fact, also be the driving force for the difference in divergence between the mt and nuclear genomes within this species. Interestingly, it has been previously demonstrated that the environment an organism inhabits can influence mitonuclear gene complexes. Furthermore, there is evidence that changes in temperature can result in selective forces acting on gene products with disparate thermal properties (Dowling et al. 2008). Additionally, discrepancies in the reproductive cycle of these species could also have an impact on mt as well as nuclear genome evolution. Nearly all natural isolates of *L. thermotolerans* are haploid, whereas many *L. kluyveri* strains appear to be diploid or even occasionally triploid (data not shown). The lack of data concerning intraspecific diversity of mt genomes among yeast lineages is apparent. Our study revealed that evolution of the mt genomes in *L. thermotoleran,* and *L. kluyveri* is clearly different and indicates a much lower mutation rate or dramatically stronger purifying selection in *L. thermotolerans*. In the future, a better understanding of the forces driving these types of selective pressures will potentially reveal how closely related lineages diverge and evolve over time.

## Supplementary Material

Supplementary tables S1–S3 and figures S1 and S2 are available at *Genome Biology and Evolution* online (http://www.gbe.oxfordjournals.org/).

Supplementary Data
