# Supplementary material for: Population Genomic Analysis Reveals Highly Conserved Mitochondrial Genomes in the Yeast Species Lachancea thermotolerans
Source: Genome Biol Evol. 2014 Sep 11;6(10):2586–94. doi: 10.1093/gbe/evu203 (PMC4224330; doi:10.1093/gbe/evu203)
Supplement: Supplementary Data [file supp_6_10_2586__index.html]

Population Genomic Analysis Reveals Highly Conserved Mitochondrial Genomes in the Yeast Species Lachancea thermotolerans — Population Genomic Analysis Reveals Highly Conserved Mitochondrial Genomes in the Yeast Species Lachancea thermotolerans — Supplementary Data 

# Population Genomic Analysis Reveals Highly Conserved Mitochondrial Genomes in the Yeast Species *Lachancea thermotolerans*

## Supplementary Data

files

**Files in this Data Supplement:**

- Supplementary Data - pdf file
