# Supplementary material for: Population Genomic Analysis Reveals Highly Conserved Mitochondrial Genomes in the Yeast Species Lachancea thermotolerans
Source: Genome Biol Evol. 2014 Sep 11;6(10):2586–94. doi: 10.1093/gbe/evu203 (PMC4224330; doi:10.1093/gbe/evu203)
Supplement: Supplementary Data [file supp_evu203_SuppInfo.pdf]

Figure S1.

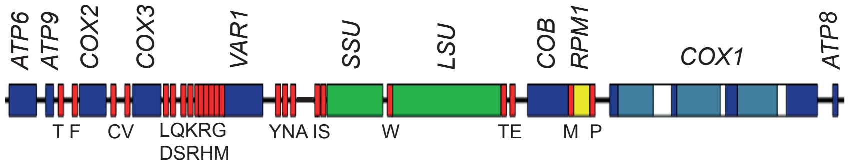

Mitochondrial genome map of *L. thermotolerans*.

The 35 genes encoded are found in both the *L. kluyveri* and by *L. thermotolerans* genomes. The protein-coding (dark blue), tRNA (red) and rRNA (green) genes are represented in addition to *RPM1* (yellow) and the coding (light blue) and noncoding introns (white). There is at least one tRNA for each amino acid, which is indicated by the single letter code below the figure. The intergenic regions of *L. thermotolerans* are small and compact (24.2%) in comparison to those in *L. kluyveri* (41%).

Figure S2.

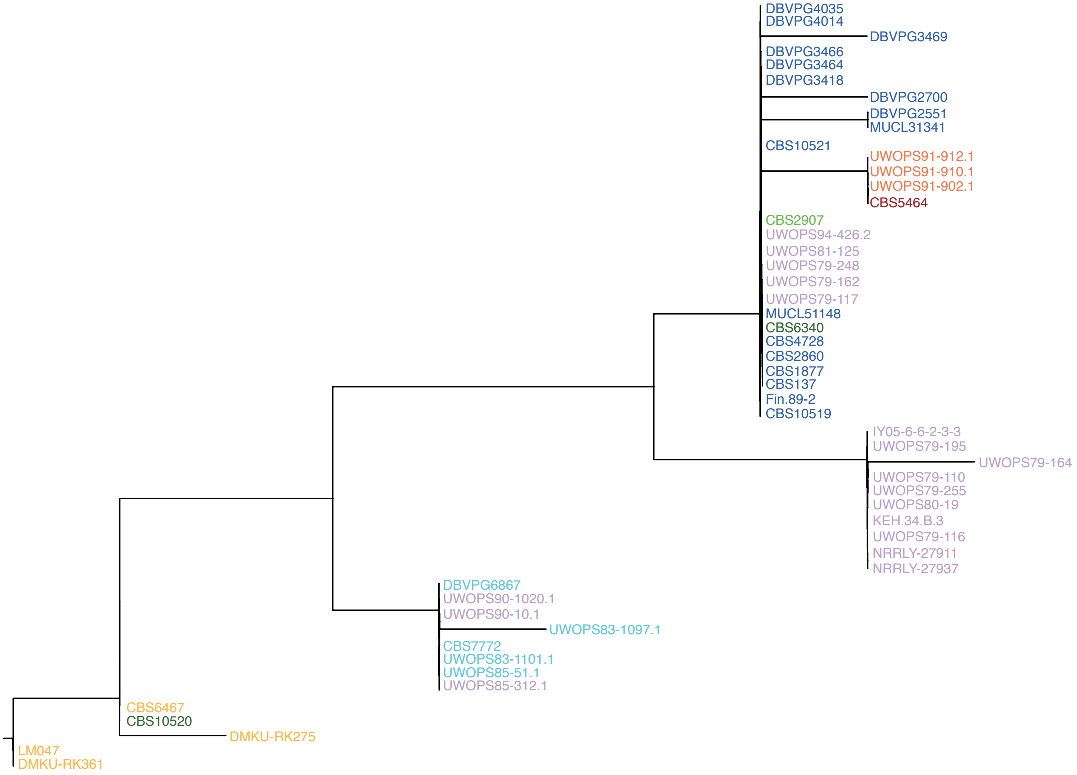

Phylogenetic tree constructed with PhyML using the *VAR1* gene, using a total of 1,125 bp. The phylogeny generally reflects the same clustering found with the concatenated gene set from all coding mt genes.

Table S1. List of the *L. thermotolerans* isolates used in this study.

Strain	Geographical Origin	Ecological Origin
CBS10519	Ukraine	exsudate of *Quercus sp.*
CBS10520	Russia	exsudate of *Quercus mongolica*
CBS10521	Finland	exsudate of *Quercus sp.*
CBS137	Netherlands	date
CBS1877	Italy	grape
CBS2860	Sardinia	grape must
CBS2907	South Africa	soil
CBS4728	Czechoslovakia	grape
CBS5464	Australia	cotton seed
CBS6340T	Russia	mirabelle-plum conserve
CBS6467	Japan	Tree exudate
CBS7772	Brazil	Uca sp. (fiddler crab)
MUCL31341	Italy	wine
UWOPS 79-110	Ontario, CA	Black knot, *Prunus virginiana*
UWOPS 79-116	Pinery, CA	Black knot, *Prunus virginiana*
UWOPS 79-117	Pinery, Ontario, CA	Black knot, *Prunus virginiana*
UWOPS 79-162	Melbourne, Ontario, CA	Black knot, *Quercus rubra*
UWOPS 79-164	Melbourne, Ontario, CA	Black knot, *Prunus virginiana*
UWOPS 79-195	Melbourne, Ontario, CA	Black knot, *Prunus virginiana*
UWOPS 79-248	Ontario, CA	Frass, Birch
UWOPS 79-255	Ontario, CA	Black knot, *Prunus pumila*
UWOPS 80-19	Ontario, CA	Black knot, *Prunus virginiana*
UWOPS 81-125	Melbourne, Ontario, CA	Gall, *Quercus* sp
UWOPS 83-1097.1	Cayman Brac, Cayman Is	*Gitona americana*
UWOPS 83-1101.1	Cayman Brac, Cayman Is	*Gitona americana*
UWOPS 85-51.1	Big Pine Key, Florida, USA	*Opuntia cubensis*
UWOPS 85-312.1	Tucson, Arizona, USA	*Drosophila carbonaria*
UWOPS 90-10.1	Shroud Cay, Exumas Cays, Bahamas	Columnar cactus fruit
UWOPS 90-1020.1	Shroud Cay, Exumas Cays, Bahamas	coco plum
UWOPS 91-902.1	Saddle Rd Park Hawaii Is, USA	flux (white), *Myoporum*
UWOPS 91-910.1	Saddle Rd Park Hawaii Is, USA	flux (pink), *Myoporum*
UWOPS 91-912.1	Saddle Rd Park Hawaii Is, USA	flux (white), *Myoporum*
UWOPS 94-426.2	Tequila, Jalisco, MX	distillery, agave must

KEH.34.B.3	Ste. Genevieve, Missouri, USA	chaumette vineyard fermentation
DMKU-RK275	Thailand	rice leaves
DMKU-RK361	Nong Muang, Thailand	sugarcane leaves
LM047	Khoa ito waterfall, Thailand	leaves
NRRL Y-27937	Los Angeles, USA	surface of male *Chauliodes rastricornis*
NRRL Y-27911	Los Angeles, USA	gut of male *Chauliodes rastricornis*
IY 05-6-6-2-3-3	Los Angeles, USA	gut of female *Chauliodes rastricornis*
DBVPG 2551	Piemonte, Italy	barbera wine
DBVPG 2700	Campo de Santiago, La Mancha, Spain	airen grapes
DBVPG 3418	Italy	milk
DBVPG 3464	Valdepenas, La Mancha, Spain	grapes
DBVPG 3466	La Encomienda, La Mancha, Spain	grapes
DBVPG 3469	Manzanares, La Mancha, Spain	grapes
DBVPG 4014	Italy	caverns
DBVPG 4035	ex Yugoslavia	grape must
DBVPG 6867	Brazil	*Pilosocereus arrabidae*
Fin. 89-2	Finland	exudate of *Quercus* sp.

Table S2. List of the 50 genes from the nuclear genomes used in the construction of the phylogenetic trees for *L. thermotolerans* and *L. kluyveri*. The positions of all genes in the concatenated alignment are indicated.

L. thermotolerans genes	Position in the alignment*	Orthologous gene in *L. kluyveri*	Position in the alignment **
KLTH0C11550g	1 to 1197	SAKL0E00814g	1 to 1197
KLTH0B01386g	1198 to 1971	SAKL0G14652g	1198 to 1983
KLTH0G00814g	1972 to 2859	SAKL0F00924g	1984 to 2871
KLTH0F05390g	2860 to 3957	SAKL0C06050g	2872 to 4092
KLTH0F18898g	3958 to 4373	SAKL0C12804g	4093 to 4509
KLTH0B04334g	4374 to 5282	SAKL0B12034g	4510 to 5430
KLTH0E12232g	5283 to 7730	SAKL0H10274g	5431 to 8085
KLTH0C03146g	7731 to 9236	SAKL0D12056g	8086 to 9567
KLTH0D08822g	9237 to 10037	SAKL0B11440g	9568 to 10302
KLTH0D08756g	10038 to 10601	SAKL0B11374g	10303 to 10896
KLTH0G05280g	10602 to 10874	SAKL0G06468g	10897 to 11193
KLTH0E15752g	10875 to 11702	SAKL0C08118g	11194 to 12051
KLTH0F10912g	11703 to 12287	SAKL0B09108g	12052 to 12642
KLTH0F14278g	12288 to 13949	SAKL0F03498g	12643 to 14241
KLTH0D06512g	13950 to 14177	SAKL0A09174g	14242 to 14469
KLTH0C09636g	14178 to 15986	SAKL0C09592g	14470 to 16338
KLTH0E08030g	15987 to 16847	SAKL0H22264g	16339 to 17202
KLTH0C04554g	16848 to 21128	SAKL0D02222g	17203 to 21480
KLTH0E09922g	21129 to 24488	SAKL0H12958g	21481 to 24870
KLTH0G05368g	24489 to 25817	SAKL0G06556g	24871 to 26202
KLTH0G12474g	25818 to 26507	SAKL0H15752g	26203 to 26889
KLTH0G14586g	26508 to 27458	SAKL0H18084g	26890 to 27861
KLTH0F03102g	27459 to 28697	SAKL0C03454g	27862 to 29100
KLTH0E02420g	28698 to 30902	SAKL0G07326g	29101 to 31314
KLTH0G03872g	30903 to 31133	SAKL0G05038g	31315 to 31545
KLTH0B08030g	31134 to 31550	SAKL0E13904g	31546 to 32016
KLTH0G09856g	31551 to 33623	SAKL0E08250g	32017 to 34080
KLTH0F01540g	33624 to 35381	SAKL0C01606g	34081 to 35931
KLTH0G02640g	35382 to 36710	SAKL0G03806g	35932 to 37275
KLTH0G09108g	36711 to 39059	SAKL0E08998g	37276 to 39747
KLTH0E01980g	39060 to 39818	SAKL0F14432g	39748 to 40506
KLTH0E02904g	39819 to 40616	SAKL0G07810g	40507 to 41322
KLTH0G18326g	40617 to 41426	SAKL0B01518g	41323 to 42120
KLTH0H06754g	41427 to 42335	SAKL0A07282g	42121 to 42897
KLTH0D06446g	42336 to 43085	SAKL0A09020g	42898 to 43641
KLTH0H14256g	43086 to 46880	SAKL0D11132g	43642 to 47781
KLTH0G03102g	46881 to 47165	SAKL0G04224g	47782 to 48066

KLTH0G03036g	47166 to 48464	SAKL0G04158g	48067 to 49359
KLTH0C10054g	48465 to 49373	SAKL0C09966g	49360 to 50493
KLTH0F19030g	49374 to 50327	SAKL0C12958g	50494 to 51594
KLTH0F19272g	50328 to 51762	SAKL0C13244g	51595 to 52803
KLTH0C02002g	51763 to 52569	SAKL0G15202g	52804 to 53616
KLTH0H00880g	52570 to 53826	SAKL0H02464g	53617 to 54867
KLTH0E07590g	53827 to 54579	SAKL0H21692g	54868 to 55650
KLTH0E03652g	54580 to 57024	SAKL0G08250g	55651 to 58146
KLTH0E12936g	57025 to 58659	SAKL0H09658g	58147 to 59679
KLTH0B05280g	58660 to 61620	SAKL0E11044g	59680 to 62745
KLTH0E10978g	61621 to 64935	SAKL0H11880g	62746 to 66375
KLTH0F10758g	64936 to 65805	SAKL0B09240g	66376 to 66900
KLTH0H09504g	65806 to 66358	SAKL0H24112g	66901 to 67456

*   the sequence alignment is provided as a supplementary file (concatAliLath.fasta)

**   the sequence alignment is provided as a supplementary file (concatAliLakl.fasta)

Table S3. The number of single nucleotide polymorphisms (SNPs) and indels found in different regions of the *L. thermotolerans* genomes used in this study.

Strain	SNPs					Indels				
	total #	in CDS	in intergenic	in intron	in rRNA	total #	in CDS	in intergenic	in intron	in rRNA
CBS10519	50	2	27	13	7	3	0	2	1	0
CBS10520	279	15	154	72	38	4	0	4	0	0
CBS10521	49	3	27	15	4	0	0	0	0	0
CBS137	1	0	1	0	0	0	0	0	0	0
CBS1877	2	0	1	1	0	11	0	8	1	2
CBS2860	20	0	14	4	2	1	0	1	0	0
CBS4728	54	1	34	13	6	5	0	3	1	1
CBS5464	56	2	31	16	7	1	0	1	0	0
CBS6340[T]	2	0	1	0	1	0	0	0	0	0
CBS6467	280	13	152	76	39	8	0	8	0	0
CBS7772	275	12	120	106	36	2	0	1	1	0
MUCL31341	46	3	25	16	2	0	0	0	0	0
UWOPS_79-110	211	8	88	92	23	3	0	2	1	0
UWOPS_79-117	47	1	28	14	4	4	0	3	1	0
UWOPS_79-162	48	1	30	13	4	3	0	2	1	0
UWOPS_79-248	50	1	31	14	4	4	0	4	0	0
UWOPS_79-255	215	8	90	94	23	1	0	1	0	0
UWOPS_80-19	208	8	82	94	24	3	0	3	0	0
UWOPS_81-125	44	1	26	13	4	7	0	6	1	0
UWOPS_83-1097.1	259	13	118	97	31	6	0	4	1	1
UWOPS_83-1101.1	259	12	114	102	31	13	0	13	0	0
UWOPS_85-51.1	287	12	130	107	37	2	0	0	2	0
UWOPS_85-312.1	292	14	123	114	40	1	0	1	0	0
UWOPS_94-426.2	21	0	15	4	2	1	0	1	0	0

KEH.34.B.3	204	8	84	89	23	7	0	7	0	0
DMKU-RK275	273	15	143	72	43	10	0	10	0	0
DMKU-RK361	286	15	158	75	38	6	0	6	0	0
LM047	267	15	148	71	33	11	0	10	0	1
NRRL_Y-27937	192	9	74	85	24	6	0	6	0	0
NRRL_Y-27911	191	8	74	86	23	3	0	3	0	0
CBS2907	50	1	30	16	3	1	0	1	0	0
UWOPS_79-116	223	8	93	98	24	0	0	0	0	0
UWOPS_79-164	215	9	89	94	23	0	0	0	0	0
UWOPS_79-195	212	8	88	93	23	3	0	3	0	0
UWOPS_90-10.1	287	13	125	110	39	0	0	0	0	0
UWOPS_90-1020.1	284	13	125	106	40	1	0	1	0	0
UWOPS_91-902.1	58	2	34	15	7	2	0	2	0	0
UWOPS_91-910.1	59	2	35	15	7	0	0	0	0	0
UWOPS_91-912.1	61	2	36	16	7	0	0	0	0	0
IY_05-6-6-2-3-3	206	8	70	102	26	7	0	7	0	0
DBVPG_2551	49	3	28	16	2	2	0	2	0	0
DBVPG_2700	83	10	45	20	8	16	0	11	0	5
DBVPG_3418	2	0	1	1	0	0	0	0	0	0
DBVPG_3464	5	0	4	1	0	0	0	0	0	0
DBVPG_3466	3	0	2	1	0	12	0	9	0	2
DBVPG_3469	46	3	22	15	6	30	0	21	2	7
DBVPG_4014	5	1	3	1	0	1	0	1	0	0
DBVPG_4035	24	0	19	3	2	1	0	1	0	0
DBVPG_6867	269	12	114	105	37	9	0	8	1	0
Fin.89-2	49	3	27	15	4	1	0	1	0	0
